# Ability of Pit and Fissure Sealant-containing Amorphous Calcium Phosphate to inhibit Enamel Demineralization

**DOI:** 10.5005/jp-journals-10005-1325

**Published:** 2016-04-22

**Authors:** Feda I Zawaideh, Arwa I Owais, Wasan Kawaja

**Affiliations:** 1 Assistant Professor, Department of Preventive Dentistry, Jordan University of Science and Technology, Irbid, Jordan; 2 Associate Professor, Department of Preventive Dentistry, Jordan University of Science and Technology, Irbid, Jordan; 3 Postgraduate Student, Department of Preventive Dentistry, Jordan University of Science and Technology, Irbid, Jordan

**Keywords:** Aegis®, Amorphous calcium phosphate, Demi-neralization, Pit and fissure sealant, Surface microhardness.

## Abstract

**Aim:** To evaluate the effect of amorphous calcium phosphate (ACP)-containing pit and fissure sealant on inhibition of enamel demineralization *in vitro.*

**Materials and methods:** Enamel specimens (n = 75) were prepared using freshly extracted noncarious human third molars. Box-shaped cavities (8 × 2 × 2 mm) on the buccal or lingual surfaces were prepared and restored with resin-based sealant (Concise™), ACP-containing sealant (Aegis®) or fluoride-containing sealant (Conseal-F™). The samples were acid challenged in a demineralizing solution of 50 mmol/l lactic acid at pH 5.0 for 4 days. The change in enamel microhardness (ASuH) was calculated. Data were analyzed using one-way analysis of variance (ANOVA) and Tukey’s *post hoc* test.

**Results:** The mean SuH_0_ (±SD) (in Vicker’s unit) prior to the acid challenge was: Concise™ (318.83 ± 33.86), Aegis® (331.03 ± 21.52), Conseal-F™ (310.12 ± 34.31). Following the acid challenge, the values dropped in all groups and ASuH (±SD) values were 269.17 ± 47.49, 151.39 ± 23.96 and 175.79 ± 32.39 respectively.

**Conclusion:** The ACP-containing pit and fissure sealant has the potential to inhibit enamel demineralization.

**How to cite this article:** Zawaideh FI, Owais AI, Kawaja W. Ability of pit and fissure sealant-containing amorphous calcium phosphate to inhibit enamel demineralization. Int J Clin Pediatr Dent 2016;9(1):10-14.

## INTRODUCTION

Amorphous calcium phosphate (ACP) is a precursor to the formation of hydroxyapatite (HAP), which is the final, stable product in the precipitation of the calcium and phosphate ions from neutral or basic solutions. Amorphous calcium phosphate has shown anti-cariogenic properties with remineralization potential.^1,2^ Amorphous calcium phosphate-containing bioactive materials stimulate mineral growth by increasing the calcium and phosphate concentrations within the lesion, especially in acidic oral environment, to levels above those existing in ambient oral fluids, thereby shifting the thermodynamic driving forces of the solution toward the formation of apatite. Amorphous calcium phosphate is believed to maintain these supersaturation conditions over extended periods of time.^3^

Amorphous calcium phosphate has both preventive and restorative material properties that justify its use in dental cements, sealants, composites and orthodontic adhesives.^1,4^ Amorphous calcium phosphate-filled composite resins have been shown to repair 71% of the lost mineral content of decalcified teeth.^1^

A new pit and fissure sealant containing ACP (Aegis® pit and fissure sealant) has been marketed for use as a light-cured sealant with similar properties to previously used resins. These materials are encouraging the formation of HAP, which can be used by the tooth for remineralization. The manufacturers claim that it has the ability to release calcium and phosphate ions and remineralize tooth structure by enhancing the tooth’s natural repair mechanism. Silva et al^2^ have demonstrated *in situ* the remineralization of artificially induced caries lesions by Aegis® and Choudhary et al^5^ demonstrated the remineralization potential of Aegis® using scanning electron microscope (SEM). Alsaffar et al^6^ demonstrated that Aegis® may provide some protective effect on demineralization of adjacent enamel measuring the amount of mineral loss. However, it was not superior to fluoride-releasing sealants.

The aim of this study is to evaluate the effect of incorporating ACP into pit and fissure sealant on inhibition of enamel demineralization and compare it with fluoride-releasing sealant using enamel microhardness tester.

## MATERIALS AND METHODS

Ethical approval for using extracted teeth was obtained from the Institutional Review Board of Jordan University of Science and Technology (JUST). Freshly extracted non-carious third molars (n=38) were collected from the dental clinics at JUST and stored in 0.1% thymol, to inhibit bacterial growth and were used within 4 months of extraction.

The roots of the teeth were removed perpendicular to the long axis of the tooth with a 0.3 mm thick diamond blade (Struers, Copenhagen, Denmark). Sound, relatively plain buccal and lingual surfaces free of cracks, stains and hypomineralized areas were selected. In order to remove the fluoride-rich surface enamel layer and to obtain a relatively flat surface, the buccal and lingual aspects of each tooth were polished with a series of aluminum oxide soflex disks (coarse, medium, fine, and superfine grit; 3M ESPE Dental Products, St Paul, MN, USA). Box-shaped cavities, 8 mm long × 2 mm wide × 2 mm deep, were prepared between the middle and cervical thirds of the buccal and lingual surfaces of each tooth using a flat fissure tungsten carbide bur 1 mm in diameter in a high-speed handpiece with air-water coolant. The cavities were prepared and finished with a slow-speed steel flat fissure bur 0.9 mm in diameter to create a cavo-surface angle as close as possible to 90°. Each crown was then sectioned into two halves in a mesiodistal direction to produce two enamel samples (buccal and lingual) (hereafter termed specimens) and stored in distilled water.

The specimens were randomly assigned into three groups one control group and two experimental groups. In group 1 (G1) (n = 25), cavities were filled with conventional resin-based sealant (control group) (Concise™ - opaque white; 3M ESPE Co. Ltd.). In group 2. (G2) (n = 25), cavities were filled with sealant-containing ACP (Aegis® - opaque white; Bosworth Co. Ltd.) and, in group 3 (G3) (n = 25), cavities were filled with resin-based sealant-containing fluoride (Conseal-F™; SDI). Each sealant material was used strictly according to their manufacturers’ instructions.

Two coats of acid-resistant nail varnish were applied to the tooth surface, leaving a window not less than approximately 1 mm wide surrounding the occlusal margin. Specimens were mounted on aluminum cylinders and surface microhardness was measured by surface microhardness tester (Matsuzawa Seiki tester) in Vickers’ units by applying 25 gm of force for 5 seconds. The hardness was measured on three different locations, with 100 μm spacing and then the average of the three readings was calculated to get the baseline surface microhardness value (baseline: SμH_0_).

Each specimen was then stored in individual plastic containers containing 40 ml of acid buffer solution consisting of 50 mmol/l lactic acid adjusted to pH 5.0. The teeth were stored in the demineralizing solution for 4 days at 37°C without refreshing the solution. After 4 days, the teeth were removed from the solution, rinsed with distilled water and microhardness measurements were recorded in the same protocol described for the baseline measurement above (final: SμH1).

**Table 1:** **Table 1:** Surface microhardness (S|jH) in Vicker’s unit for different fissure sealant treatments before and after the acid challenge

*Groups*		*Means of SμH_0_ ±* * std. deviation*		*SμH_0_ range*		p-value*		*Groups*		*Means of SμH_1_ ±* * std. deviation*		*SμH_1_ range*		p-value*	
G1 (n = 24)		318.83 ± 33.86		274.3-401.1		0.058		G1 (n = 24)		49.66 ± 29.86		16.7-126.4 0.000
G2 (n = 25)		331.03 ± 21.52		289.2-372.6				G2 (n = 25)		179.63 ± 23.36		131.5-214.1			
G3 (n = 25)		310.12 ± 34.31		231.7-372.2				G3 (n = 25)		134.52 ± 19.1		95.8-180.0		

*Significant when p < 0.05

## STATISTICAL ANALYSIS

Descriptive statistics were prepared for surface micro-hardness values for each group; means were compared using Statistical Package for the Social Science (SPSS) version 19.0 for Windows (SPSS Inc., Chicago, USA). Assumptions of normality and equal variance were verified and one-way analysis of variance (ANOVA) comparison of means was performed at a significance level of 0.05. When ANOVA revealed a difference between groups, Tukey’s *post hoc* test was performed to compare between the changes in microhardness by acid for the three groups.

## RESULTS

One specimen in group 1 failed following the acid exposure resulting in 24 specimens available in this group and 25 specimens in each of groups 2 and 3.

The mean (±SD) surface microhardness (SμH_0_) values of the specimens for the three different treatment groups before acid challenge are shown in Table 1. There were no statistically significant differences between the surface microhardness means of the three groups before the acid challenge (p = 0.058).

Following the acid challenge, the mean surface microhardness (SμH_1_) values of the three groups dropped significantly (Table 1). Group 2 had the highest mean SμH of 179.63 ± 23.36 (Vicker’s unit reaching up to triple that of group 1). Statistically significant differences existed in the mean surface microhardness between the three groups following the acid challenge (p = 0.000).

The mean SμH difference (SμH_0_ - SμH_1_) between pre-and postacid challenge values was calculated (Table 2). Measurements following the acid application were significantly lower for all fissure sealant groups than those prior to the acid application. The amount of difference was highest in group 1 followed by group 3. A statistically significant difference existed between the three groups withp = 0.000.

Multiple comparisons of the mean SμH difference among the three groups detected a statistically significant relationship (p = 0.000) between groups 1 to 3. There was also a significant difference between the two treatment groups 2 and 3 (p = 0.019).

**Table 2:** **Table 2:** Mean microhardness change (in Vicker’s unit) by application of acid for different fissure sealant groups

*Mean SμH difference within the groups*										*Mean SμH difference between the groups*									
														*95% confidence interval*					
*Groups*		*Mean SμH* *difference ±* *std. deviation*		*Min.*		*Max.*		p-value*		*Groups*		*Mean SμH* *difference ±* *std. std. error*		*Lower* * bound*		*Upper* * bound*		p-value*	
G1 (n = 24)		269.17 ± 47.49		168.64		379.33		0.000		G1-G2		117.78 ± 10.22		97.40		138.16		0.000	
G2 (n = 25)		151.39 ± 23.96		99.36		201.39				G1-G3		93.50 ± 10.22		73.12		113.89		0.000	
G3 (n = 25)		175.79 ± 32.39		110.09		224.85				G2-G3		24.28 ± 10.12		4.10		44.45		0.019	

*Statistically significant if p < 0.05

## DISCUSSION

### Effect of ACP-containing Fissure Sealant on inhibition of Demineralization

Amorphous calcium phosphate-containing fissure sealant was shown to be effective in inhibition of enamel demineralization. This is in agreement with the previous *in vitro* and *in situ* studies.^2,5,6^ Silva et al^2^ demonstrated *in situ* that sealants containing ACP and/or fluoride presented a higher remineralizing capacity than that of the control group, but the sealants containing ACP provided either more efficient or similar remineralization, than the other sealants containing fluoride. They were able to promote remineralization of artificially induced carious lesions on the smooth enamel surfaces.^2^

A recent *in vitro* study was conducted by Choudhary et al^5^ to evaluate the remineralization potential of ACP and fluoride-containing pit and fissure sealants using SEM. After pH cycling, qualitative changes at the tooth surface and sealant interface were examined. The presence of white zone at the interface was considered positive for remineralization. Both ACP-containing and fluoride-containing groups showed white zone at the tooth surface-sealant interface. However, the white zone in the ACP group had irregular, granular or globular zone evident in few areas, while granular or globular zones were not evident in the fluoride-releasing group. They concluded that both ACP- and fluoride-containing sealants had the potential for remineralization. The release of ACP molecules in the ACP group and formation of fluorapatite in the fluoride group were responsible for the remineralization.^5^

### Effect of Fluoride-releasing Fissure Sealant on inhibition of Demineralization

The effectiveness of fluoride released from various dental materials in caries prevention by promoting remineralization and inhibiting demineralization of enamel has been widely demonstrated.^7,8^

However, clinical studies exhibited conflicting data as to whether or not these fluoride-containing materials significantly prevent or inhibit secondary caries and affect the growth of cariogenic bacteria compared with non-fluoridated materials.^9^ A study conducted by Vatanatham et al^9^ showed that the mineral loss of incipient enamel carious lesions sealed with fluoride-containing sealants was not significantly different from those of nonfluoride-containing sealants. Short- and long-term fluoride releases from restoratives are related to their matrices, setting mechanisms and fluoride content and depend on several environmental conditions.^9^

In our study, Conseal-F™ provided increased demine-ralization inhibition compared to conventional sealant containing no fluoride. This is in accordance with many *in vitro* studies.^6,10^

Garçía-Godoy et al^11^ conducted a study to compare the amounts and patterns of fluoride release from five different fluoride-containing pit and fissure sealants. They found that all fluoride-containing sealants released fluoride in the same pattern throughout the test period of 30 days; i.e., the greatest amount of fluoride was released in the first 24 hours after mixing, fell sharply on the second day and decreased slowly in the final days.^11^

Fluoride released from glass ionomer cement (GIC)-based dental materials was significantly more than fluoride released from resin-based materials containing fluoride.^12^ Studies on fluoride release from pit and fissure sealants demonstrated the same findings. Salar et al^10^ found that incorporation of fluoride into sealants provided increased demineralization inhibition compared with a conventional sealant containing no fluoride, but less than that shown by a glass ionomer sealant.^10^

An *in vitro* study conducted by Kantovitz et al^13^ aimed to test the enamel mineral loss inhibition ability at the enamel/sealant interface by fluoride-containing (both GIC- and resin-based) and nonfluoride-containing sealants. The results demonstrated that resin sealant did not prevent enamel mineral loss, contrary to GIC, which showed the highest capacity for fluoride release. They concluded that the presence of fluoride in a material’s composition *per se* does not indicate its capability to prevent the development of enamel caries-like lesions.^13^

### Comparison between Aegis® and Conseal-F™ in inhibition of Enamel Demineralization

Similar to previous studies, our results demonstrated that both agents were found to be effective in the prevention of enamel demineralization.^2,5,6^ However, in this study, the results showed that Aegis® had higher surface microhardness mean values than Conseal-F™ after acid challenge and lower mean difference between pre- and postacid challenge values.

Amorphous calcium phosphate-containing materials have many advantages over fluoride-releasing materials: (1) Results of quantitative microradiographic study demonstrated that remineralization of subsurface enamel lesions by fluoride is a self-limiting surface phenomenon that prevents penetration of ions into the depth of the lesion. Mineral deposition occurred in the initial 30% of the lesion only. Thereafter, it occludes the surface pores and limits the repair of the remainder of the lesion. On the contrary, ACP-containing materials deliver minerals deeper into the lesion and deposit significantly more mineral overall than fluoride-releasing materials.^3^ (2) The effect of the fluoride ion may be limited by the availability of the Ca and PO_4_ in the plaque and saliva in the oral cavity, whereas the high solubility of ACP makes it dissolve as Ca and PO4 ions, thereby supersaturating the area locally and promoting the formation of HAP.^14^ (3) ACP-containing materials are "smart" materials, i.e., they release Ca and PO_4_ ions only when the pH drops below 5.5 and ceases when the pH rises,^15^ whereas the pattern of fluoride release from dental material tends to show the greatest amount of fluoride release in the first 24 hours after mixing and then fall sharply on the second day and decreases slowly over the following days.^11^

## CONCLUSION

Within the limitations of this *in vitro* study, the following conclusions can be drawn:

- The ACP-containing pit and fissure sealant demonstrated the highest inhibition in enamel demineralization when compared with the other types of fissure sealants.
- Fluoride-containing sealants may provide some protective effect on demineralization of adjacent enamel compared with conventional sealants without fluoride.
